# Improving reliability estimation in cognitive diagnosis modeling

**DOI:** 10.3758/s13428-022-01967-5

**Published:** 2022-09-20

**Authors:** Rodrigo Schames Kreitchmann, Jimmy de la Torre, Miguel A. Sorrel, Pablo Nájera, Francisco J. Abad

**Affiliations:** 1 Department of Social Psychology and Methodology, Faculty of Psychology, Universidad Autónoma de Madrid, Calle Iván Pavlov, 6, Ciudad Universitaria de Cantoblanco, 28049 Madrid, Spain; 2 School of Science and Technology, IE University, Paseo de la Castellana, 259, Madrid, Spain; 3 Faculty of Education, The University of Hong Kong, Pokfulam Road, Pok Fu Lam, Hong Kong

**Keywords:** Cognitive diagnosis, Diagnostic classification, Reliability, Classification accuracy, Multiple imputation

## Abstract

Cognitive diagnosis models (CDMs) are used in educational, clinical, or personnel selection settings to classify respondents with respect to discrete attributes, identifying strengths and needs, and thus allowing to provide tailored training/treatment. As in any assessment, an accurate reliability estimation is crucial for valid score interpretations. In this sense, most CDM reliability indices are based on the posterior probabilities of the estimated attribute profiles. These posteriors are traditionally computed using point estimates for the model parameters as approximations to their populational values. If the uncertainty around these parameters is unaccounted for, the posteriors may be overly peaked, deriving into overestimated reliabilities. This article presents a multiple imputation (MI) procedure to integrate out the model parameters in the estimation of the posterior distributions, thus correcting the reliability estimation. A simulation study was conducted to compare the MI procedure with the traditional reliability estimation. Five factors were manipulated: the attribute structure, the CDM model (DINA and G-DINA), test length, sample size, and item quality. Additionally, an illustration using the *Examination for the Certificate of Proficiency in English* data was analyzed. The effect of sample size was studied by sampling subsets of subjects from the complete data. In both studies, the traditional reliability estimation systematically provided overestimated reliabilities, whereas the MI procedure offered more accurate results. Accordingly, practitioners in small educational or clinical settings should be aware that the reliability estimation using model parameter point estimates may be positively biased. *R* codes for the MI procedure are made available

Cognitive diagnosis models (CDMs) have recently gained popularity as an efficient tool for diagnostic assessment (e.g., de la Torre & Minchen, 2014; von Davier & Lee, 2019). CDMs can be viewed as a family of constrained latent class models for classifying subjects with respect to discrete, usually binary, attributes (e.g., mastery or non-mastery of a set of skills) underlying structured assessment data (de la Torre & Douglas, 2004; Templin & Henson, 2006), hence grouping them into different latent classes (i.e., attribute profiles).

Although primarily developed for evaluating student mastery and non-mastery of cognitive skills (and hence the name *cognitive diagnosis models*), CDM use has gone beyond educational settings, being applied in various domains (Sessoms & Henson, 2018). Specifically, current literature includes CDM applications not only in mathematics (e.g., Y.-H. Chen et al., 2019; Tang & Zhan, 2020), reading (e.g., George & Robitzsch, 2021), or foreign language evaluation (e.g., Dong et al., 2021; Du & Ma, 2021), but also for assessing personality (e.g., Huang, 2022; Revuelta et al., 2018), psychological disorders (e.g., de la Torre et al., 2018; Xi et al., 2020) or work and study attitudes (e.g., García et al., 2014; Sorrel et al., 2016). Additionally, CDMs are currently being implemented across heterogeneous conditions (Sessoms & Henson, 2018), with sample sizes as small as 44 (Jang et al., 2015) and up to 71,000 respondents (George & Robitzsch, 2014) , with recent simulation studies supporting the use of parametric CDM methods for sample sizes as small as 100 (e.g., Ma et al., 2022; Ma & Jiang, 2021). In fact, there is a growing trend towards implementation of CDMs with small samples (e.g., Fan et al., 2021; Tang & Zhan, 2021), as they constitute a common context for diagnostic assessment in which tailored feedback and remediation can be easily provided.

As in any other assessment type, making correct diagnostic classifications is crucial, as it can have important consequences for the respondents. For instance, in the context of educational assessment, the use of diagnostic information about the students’ strengths and weaknesses can be helpful in guiding teaching efforts and tailoring remedial instructions both at the student and classroom levels (de la Torre & Minchen, 2014; Swan & Foster, 2018; Tang & Zhan, 2021). Similarly, in clinical settings, CDMs may facilitate practitioners to refer patients to the most adequate treatment, potentially increasing its effectiveness (e.g., Xi et al., 2020). However, beyond the classification accuracy itself, obtaining precise reliability estimates is crucial for proper decision-making (American Educational Research Association [AERA] et al., 2014). In this sense, overestimated reliabilities may lead to overly confident decision-making about uncertain classifications. This, for instance, may lead to failing to provide educational support for students in need, or to refuse treatment to patients that need to be treated. In this sense, the study of the reliability estimators in CDMs is recent but extensive. For instance, Sinharay and Johnson (2019) provide a list of 21 classification precision indices at the attribute and attribute profile levels. Broadly, these estimators can be categorized as measuring: (a) the classification accuracy, or the likelihood that the estimated classification is equal to the true classification (e.g., Cui et al., 2012; Wang et al., 2015), and (b) the classification consistency, or the likelihood that two parallel forms would yield the same estimated classifications (e.g., Cui et al., 2012; Wang et al., 2015).

## Purpose of the current article

As it will be further detailed, CDM classification accuracy and consistency estimators generally rely on the posterior probability distribution of the attribute profiles, which can be obtained using the likelihood of the observed responses for each possible attribute profile, and the attribute profile distribution. Specifically, the likelihood of the data under each attribute profile is generally computed assuming the estimated model parameters as true known quantities. In this sense, CDM reliability traditionally disregards the uncertainty around these model parameter estimates. From a frequentist perspective, the estimated model parameters may diverge from the true quantities due to the sampling error, which is reflected in the sampling distribution of these parameters. From a Bayesian point of view, for finite samples, the use of parameter point estimates will produce an underestimation of the width of the posterior distributions (Tsutakawa & Johnson, 1990; Yang et al., 2012) and, consequently, an overestimation of the reliability. Beyond that, CDM item parameter estimates (i.e., correct response probability) obtained with traditional estimation (i.e., marginal maximum likelihood estimation) have been found to be biased towards the boundaries (i.e., 0 or 1) when sample sizes are small (e.g., W. Ma & Guo, 2019; W. Ma & Jiang, 2021; Vermunt & Magidson, 2004), which may be an indicator of local maximum solution or identification problems (Uebersax, 2000). The extreme estimates, in turn, will produce even more peaked posterior distributions, which will derive into overestimated reliabilities.

In summary, assuming the point estimates of the model parameters as true values for computing classification accuracy or consistency indices may provide overestimated reliability, which can lead to incorrect decisions with overconfidence. Accordingly, the purpose of this study is to provide a way to account for the uncertainty around the model parameters in CDM reliability estimation. A multiple imputation procedure is proposed to estimate the reliability indices with corrected posterior distributions by integrating out these parameters. First, a brief overview of CDMs will be made, then the main CDM reliability estimators will be presented, and finally, the multiple imputation procedure will be explained in more detail and tested in both a simulation and real data studies.

## Overview of cognitive diagnosis models

As previously mentioned, CDMs serve as an efficient tool for assessing discrete latent variables (i.e., attributes) from structured assessment data. In this sense, CDMs allow classifying respondents according to their discrete levels in each attribute, hence grouping them into different latent classes or attribute profiles. For the usual case of binary attributes, there are a total of 2^*K*^ possible attribute profiles, where *K* denotes the number of attributes measured by a test (for polytomous attributes, see J. Chen & de la Torre, 2013).

To reach this output, CDMs require three main inputs. First, the response data to the assessment items. Second, a content-specification matrix reflecting which attributes measure each item. In this matrix, referred to as *Q-matrix* (Tatsuoka, 1983), each *q-entry* (*q*_*jk*_) will receive a value of 1 or 0 depending on whether item *j* measures attribute *k* or not, respectively. The Q-matrix construction process is usually supervised by domain experts (e.g., Li & Suen, 2013; Sorrel et al., 2016), although several empirical Q-matrix estimation and validation methods have been proposed in the last years with the aim of reducing the degree of subjectivity involved in the task (e.g., de la Torre & Chiu, 2016; Nájera, Sorrel et al., 2021). The correct specification of the Q-matrix is of major importance since the presence of misspecifications can greatly disrupt the accuracy of attribute profile classifications (Gao et al., 2017; Rupp & Templin, 2008). Finally, the third key element of CDMs is the definition of the response processes or item response functions, which refers to the specific formulation of how the attributes are associated with the item responses.

A wide variety of CDMs exist, accounting for the different nature of the attributes (e.g., J. Chen & de la Torre, 2013; de la Torre, 2011) and response data (e.g., de la Torre, 2009; W. Ma & de la Torre, 2016), as well as outlining different response processes. For simplicity, this article will focus only on CDMs for binary attributes (e.g., mastery or non-mastery) and dichotomous responses. This case can be easily generalized to other contexts. CDMs can be broadly divided into general and reduced models. General models allow the estimation of all main and interaction effects between the attributes over the responses, thus allowing for a different probability of success for every latent group. The *generalized deterministic input, noisy* and *gate* model (G-DINA; de la Torre, 2011) is a commonly used general CDM in which the success probability (i.e., scoring 1) of item *j* for respondent *i* with attribute pattern **α**_*l*_ is defined by Eq. (1).

1 $$P\left({x}_{ij}=1|{\boldsymbol{\upalpha}}_{lj}^{\ast },{\boldsymbol{\updelta}}_j\right)={\delta}_{j0}+\sum_{k=1}^{K_j^{\ast }}{\delta}_{jk}{\alpha}_{lk}+\sum_{k^{\prime }=k+1}^{K_j^{\ast }}\sum_{k=1}^{K_j^{\ast }-1}{\delta}_{jk{k}^{\prime }}{\alpha}_{lk}{\alpha}_{l{k}^{\prime }}\dots +{\delta}_{j12\dots {K}_j^{\ast }}\prod_{k=1}^{K_j^{\ast }}{\alpha}_{lk},$$

where ${\boldsymbol{\alpha}}_{lj}^{\ast }$ is the reduced attribute profile *l* whose elements are relevant to solve item *j* (i.e., with Q-matrix entries of 1) and ${K}_j^{\ast }$ is the number of attributes required to solve item *j*. Additionally, **δ**_*j*_ represents the *j*^th^ item parameter vector, where *δ*_*j*0_ denotes the baseline probability of item *j*, *δ*_*jk*_ is the main effect due to *α*_*lk*_; ${\delta}_{jk{k}^{\prime }}$ is the interaction effect due to *α*_*lk*_ and ${\alpha}_{l{k}^{\prime }}$, and ${\delta}_{j12\dots {K}_j^{\ast }}$ is the interaction effect due to ${\alpha}_{l1},\dots, {\alpha}_{l{k}^{\prime }}$. Note that *α*_*lk*_ is a binary variable indicating whether the respondent masters attribute *k* (*α*_*lk*_ = 1) or not (*α*_*lk*_ = 0).

General CDMs subsume most reduced CDMs, which are more parsimonious models that restrict the attribute interactions space. Popular reduced CDMs are the conjunctive, non-compensatory *deterministic input, noisy* and *gate* model (DINA; Junker & Sijtsma, 2001), the disjunctive, compensatory *deterministic input, noisy* or *gate* model (DINO; Templin & Henson, 2006), or the *additive cognitive diagnosis model* (A-CDM; de la Torre, 2011). Among the reduced CDMs, the DINA model has received the most attention in both simulation and applied studies (Sessoms & Henson, 2018). In this model, only two parameters are estimated per item, regardless of the number of attributes measured by the item. The success probability is computed as in Eq. (2), where a success probability of *δ*_*j*0_ is expected if respondent *i* doesn’t master at least one of the attributes required by item *j* (i.e., ${\boldsymbol{\upalpha}}_{lj}^{\ast}\ne \mathbf{1}$). On the contrary, if the respondent masters all the attributes required by the item, the success probability will be ${\delta}_{j0}+{\delta}_{j12\dots {K}_j^{\ast }}$. Probabilities *δ*_*j*0_ and $1-{\delta}_{j0}+{\delta}_{j12\dots {K}_j^{\ast }}$ are also known as *guessing* (*g*_*j*_) and *slip* (*s*_*j*_) parameters, respectively. The first denotes the success probability of item *j* for the examinees that lack at least one of the attributes involved in this item, i.e., ${g}_j=P\left({x}_{ij}=1|{\boldsymbol{\upalpha}}_{lj}^{\ast}\ne \mathbf{1},{\boldsymbol{\updelta}}_j\right)$. The second defines to the probability of incorrectly answering item *j* for the respondents that master all the attributes involved in this item, i.e., ${s}_j=P\left({x}_{ij}=0|{\boldsymbol{\upalpha}}_{lj}^{\ast }=\mathbf{1},{\boldsymbol{\updelta}}_j\right)$.

2 $$P\left({x}_{ij}=1|{\boldsymbol{\upalpha}}_{lj}^{\ast },{\boldsymbol{\updelta}}_j\right)={\delta}_{j0}+{\delta}_{j12\dots {K}_j^{\ast }}\prod_{k=1}^{K_j^{\ast }}{\alpha}_{lk}$$

Outlining the appropriate response process (i.e., selecting the correct CDM) is crucial to obtain accurate attribute profile classifications (Gao et al., 2017; Sorrel et al., 2021). General CDMs are flexible, saturated models (i.e., they estimate a success probability for all the ${2}^{K_j^{\ast }}$ reduced attribute profiles for each item) that show better model fit than reduced CDMs. However, the exponential growth of the number of item parameters as a function of the complexity of the Q-matrix might pose estimation challenges whenever the sample size is not large (Oka & Okada, 2021; Sen & Cohen, 2021). This should be a lesser problem for the reduced CDMs, such as the DINA model, which only estimate two parameters per item regardless of ${K}_j^{\ast }$.

### Model parameters estimation

Under the assumption of conditional independence between the items, the likelihood function of CDMs for binary attributes and dichotomous responses is given by Eq. (3) (de la Torre, 2011).

3 $$lik\left({\mathbf{x}}_i|{\boldsymbol{\upalpha}}_l,\boldsymbol{\updelta} \right)=\prod_{j=1}^JP{\left({x}_{ij}=1|{\boldsymbol{\upalpha}}_l,{\boldsymbol{\updelta}}_j\right)}^{x_{ij}}{\left[1-P\left({x}_{ij}=1|{\boldsymbol{\upalpha}}_l,{\boldsymbol{\updelta}}_j\right)\right]}^{1-{x}_{ij}},$$

where **x**_*i*_ is the response vector of examinee *i*, **α**_*l*_ is the *l*^th^ attribute profile among the *L* = 2^*K*^ latent classes, and **δ** is the complete set of item parameters in the test. Using marginal maximum likelihood estimation (MMLE), the fittest item parameter estimates are the ones that maximize the complete data likelihood, i.e., *lik*(**X**) as in Eq. (4) (de la Torre, 2011).

4 $$lik\left(\mathbf{X}\right)=\prod_{i=1}^I\sum_{l=1}^L lik\left({\mathbf{x}}_i|{\boldsymbol{\upalpha}}_l,\boldsymbol{\updelta} \right)P\left({\boldsymbol{\upalpha}}_l\right)$$

where *P*(**α**_*l*_) denotes the prior probability of attribute profile **α**_*l*._

The MMLE is commonly implemented through the Expectation-Maximization (EM) algorithm (Dempster et al., 1977), which consists of an iterative two-step procedure. First, step E consists of updating the expectations for all *P*(**α**_*l*_), i.e., the multinomial posterior distribution of the latent profiles (also represented as **π**), based on the empirical data and assuming a set of values for $\hat{\boldsymbol{\updelta}}$. Second, step M, consists of estimating the new **δ** parameters that maximize *lik*(**X**) given the new *P*(**α**_*l*_) values. These steps are repeated until the changes in *lik*(**X**) or in the model parameter estimates are negligible. The final set of model parameters includes the values of $\hat{\boldsymbol{\updelta}}$ and $\hat{P}\left({\boldsymbol{\upalpha}}_l\right)$ upon convergence.

### Attribute profile estimation

The attribute profile estimation generally consists of the following step, once obtained the marginal maximum likelihood estimates of the model parameters, i.e., $\hat{\boldsymbol{\updelta}}$ and $\hat{P}\left({\boldsymbol{\upalpha}}_l\right)$. The maximum likelihood estimate (MLE) of $\hat{\boldsymbol{\upalpha}}$ for respondent *i* will be of class *l* for which *lik*(**x**_*i*_| **α**_*l*_, **δ**) is highest, assuming the MMLE estimates of **δ** as correct. Under a Bayesian approach, the estimated classification for each examinee is based on the expected or maximum posterior probability of each attribute profile, refer to as expected-a-posterior (EAP) or maximum-a-posterior (MAP) estimations, respectively. The posterior probability of attribute profile **α**_*l*_ for examinee *i* is numerically approximated as in Eq. (5). As a form of empirical Bayes, the MMLE estimates of all *P*(**α**_*l*_) computed in the E-step (i.e., **π**), are assumed as prior distribution of **α**_*l*_.

5 $$P\left({\boldsymbol{\upalpha}}_l|{\mathbf{x}}_i,\boldsymbol{\updelta}, \boldsymbol{\uppi} \right)=\frac{lik\left({\mathbf{x}}_i|{\boldsymbol{\upalpha}}_l,\boldsymbol{\updelta} \right)P\left({\boldsymbol{\upalpha}}_l\right)}{\sum_{l=1}^L lik\left({\mathbf{x}}_i|{\boldsymbol{\upalpha}}_l,\boldsymbol{\updelta} \right)P\left({\boldsymbol{\upalpha}}_l\right)}.$$

Whereas the MAP estimator classifies each examinee to its most probable attribute profile, the EAP estimator computes the marginal probability of mastering each attribute separately, and then determines the discrete levels of mastery and non-mastery of each attribute based on a threshold (e.g., 0.5). For simplicity, this article will only focus on the EAP estimator, although the results should be comparable with the MAP estimator. It should be noted also that MLE is equivalent to MAP with a uniform prior.

## Reliability estimation in cognitive diagnosis modeling

Either by using MLE, EAP, or MAP attribute profile estimation, CDM scores should be complemented with an estimation of the degree of certainty around those classifications. That is, the reliability of the scores should always be reported (American Educational Research Association [AERA] et al., 2014). As previously indicated, several reliability estimators exist within the CDM framework (Sinharay & Johnson, 2019). Generally, the reliability (accuracy and consistency) of CDM classifications may be quantified in two major metrics: 1) in correlation metric, as in traditional psychometrics with continuous latent variables (e.g., Johnson & Sinharay, 2020; Templin & Bradshaw, 2013), or 2) proportion metric, which fits the discrete nature of CDMs (e.g., Wang et al., 2015). Regarding the later, most of these indicators provide a way to quantify the precision of the classifications based on approximations to the cross-classification contingency table of true and estimated attributes or attribute profiles. For instance, Table 1 illustrates the cross-classification table for the mastery of the *k*^th^ attribute measured by the test, where the proportion of correct attribute classification (i.e., τ_*k*_) is defined as $P\left\{{\hat{\boldsymbol{\upalpha}}}_k\left(\mathbf{X}\right)={\boldsymbol{\upalpha}}_k\right\}$ can be broken down to τ_*k*_ = *p*_00_ + *p*_11_. A similar table could be constructed for the classification consistency, where rows would represent estimated ${\hat{\alpha}}_k$ in a parallel assessment. As an illustration, this article will limit to the estimation of the classification accuracy in the proportion metric, although the implications of this study should be largely generalizable to classification consistency estimators and to the correlation metric.

**Table 1:** Cross-classification contingency table of true and estimated mastery of the k^th^ attribute

True *α*_*k*_	Estimated ${\hat{\alpha}}_k$		Total
	0	1	
0	*p*_00_	*p*_01_	*p*_0·_
1	*p*_10_	*p*_11_	*p*_1·_
Total	*p*_·0_	*p*_·1_	1

As stated, the classification accuracy can be defined as the proportion of correct classifications, either at the attribute level (PCA) or the attribute vector level (PCV). If the true classifications were known, the computation of PCA and PCV would be straightforward just by comparing the true and estimated attribute profiles. However, since the true **α** vectors are unknown in applied settings, PCA and PCV values must be approximated using empirical estimates. In this sense, Wang et al. (2015) proposed the τ_*k*_ and τ indices as estimators of the PCA and PCV, respectively. The τ_*k*_ index for attribute *k* is calculated as in Eq. (6).

6 $${\uptau}_k=\frac{\sum_{i=1}^N{\hat{\upalpha}}_{ik}P\left({\hat{\upalpha}}_{ik}|{\mathbf{x}}_i,\boldsymbol{\updelta}, \boldsymbol{\uppi} \right)+\left(1-{\hat{\upalpha}}_{ik}\right)\left[1-P\left({\hat{\upalpha}}_{ik}|{\mathbf{x}}_i,\boldsymbol{\updelta}, \boldsymbol{\uppi} \right)\right]}{N},$$

where ${\hat{\upalpha}}_{ik}$ denotes the estimated discrete classification of respondent *i* in attribute *k* and $P\left({\hat{\upalpha}}_{ik}|{\mathbf{x}}_i,\boldsymbol{\updelta}, \boldsymbol{\uppi} \right)$ represents the marginal posterior probability for that estimated ${\hat{\upalpha}}_{ik}$ classification given the response vector **x**_*i*_, item parameters **δ**, and latent class distribution **π**. In turn, the marginal $P\left({\hat{\upalpha}}_{ik}|{\mathbf{x}}_i,\boldsymbol{\updelta}, \boldsymbol{\uppi} \right)$ is calculated as the sum of *P*(**α**_*l*_| **x**_*i*_, **δ**, **π**) for every **α**_*l*_ in which ${\alpha}_{lk}={\hat{\alpha}}_{ik}$. The τ index can be calculated as the average of the posterior probability of the attribute profiles, as in Eq. (7).

7 $$\uptau =\frac{\sum_{i=1}^NP\left({\hat{\boldsymbol{\upalpha}}}_i|{\mathbf{x}}_i,\boldsymbol{\updelta}, \boldsymbol{\uppi} \right)}{N}.$$

It can be inferred from Eqs. (6) and (7) that τ_*k*_ and τ estimators depend on the extent that the posterior probability estimates are accurate, which, in turn, depends on the likelihood estimation (Eq. 3) and, ultimately, on the precision of the model parameter estimates (i.e., $\hat{\boldsymbol{\updelta}}$ and $\hat{\boldsymbol{\uppi}}$). Specifically, assuming the point estimates of the model parameters for computing $P\left({\hat{\boldsymbol{\upalpha}}}_i|{\mathbf{x}}_i,\boldsymbol{\updelta}, \boldsymbol{\uppi} \right)$ does not account for the uncertainty around **δ** and **π** caused by the sampling errors (Tsutakawa & Johnson, 1990; Yang et al., 2012). This will derive into overly peaked likelihood and posterior distributions, which will only approximate the true distributions if $\hat{\boldsymbol{\updelta}}$ and $\hat{\boldsymbol{\uppi}}$are precise estimates of **δ** and **π**, respectively (with sample sizes approaching infinity). Specifically, the estimation of **δ** is expected mostly to depend on the sample size and model complexity (i.e., number of parameters), whereas the estimation of **π** should be affected by both sample size and test length. Additionally, if the item parameter estimates are biased towards the boundaries (as indicated by W. Ma & Guo, 2019; W. Ma & Jiang, 2021; Vermunt & Magidson, 2004) due to local maxima or to identification problems (Uebersax, 2000), the likelihood and posterior distributions will be even more peaked. Accordingly, as can be inferred from Eqs. (6) and (7), if $P\left({\hat{\boldsymbol{\upalpha}}}_i|{\mathbf{x}}_i,\boldsymbol{\updelta}, \boldsymbol{\uppi} \right)$, and consequently $P\left({\hat{\upalpha}}_{ik}|{\mathbf{x}}_i,\boldsymbol{\updelta}, \boldsymbol{\uppi} \right)$, are overly peaked, τ_*k*_ and τ will be positively biased. The same will occur for most, if not all, available classification accuracy indices. Although it may not affect the examinees’ classification, as it may not change the ordering of the probabilities associated with each classification profile, it generates a false confidence about the reliability of the resulting classifications. In other words, it is very likely that, in small sample settings, a practitioner might wrongly conclude that the classifications obtained with a CDM application are accurate.

## Correcting reliability estimation for model parameter uncertainty

Aiming to obtain more accurate reliability estimators, we propose a method to better estimate the posterior probabilities, *P*(**α**_*l*_| **x**_*i*_, **δ**, **π**) and *P*(α_*lk*_| **x**_*i*_, **δ**, **π**), by accounting for the uncertainty around the estimation of **δ** and **π**. Let **ϑ** denote the complete vector of model parameters, i.e., **ϑ** ***=*** (**δ**, **π**)^T^. Analytically, if the sampling distribution of **ϑ** is known, **ϑ** can be integrated out of the posteriors (Eq. 8), as outlined by Tsutakawa and Johnson (1990) in the context of IRT modeling.

8 $$P\left({\boldsymbol{\upalpha}}_l|{\mathbf{x}}_i\right)=\int P\left({\boldsymbol{\upalpha}}_l|{\mathbf{x}}_i,\boldsymbol{\upvartheta} \right)P\left(\boldsymbol{\upvartheta} |\mathbf{X}\right)d\boldsymbol{\upvartheta}$$

**Fig. 1:**
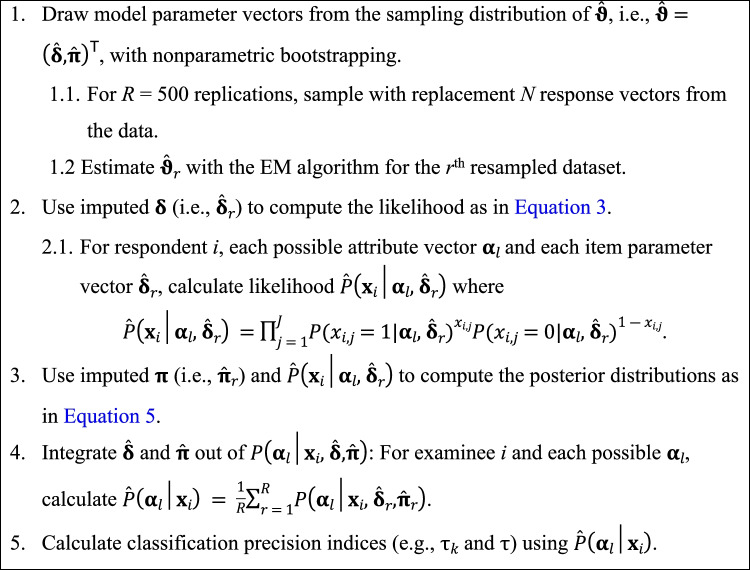
Schematic description of the multiple imputation procedure

As proposed by Yang et al. (2012), a multiple imputation (MI) approximation can be used to integrate **ϑ** out of *P*(**α**_*l*_| **x**_*i*_, **ϑ**) by 1) calculating the likelihood of **x**_*i*_, *P*(**x**_*i*_| **α**_*l*_, **δ**) with *R* imputed ${\hat{\boldsymbol{\updelta}}}_r$ vectors drawn from the sampling distribution of **ϑ**, 2) imputing *R* random ${\hat{\boldsymbol{\uppi}}}_r$ vectors drawn from the sampling distribution of **ϑ** to calculate the posteriors, and 3) marginalizing over both **δ** and **π**. Since the occurrence of boundary parameter estimates may generate numerical difficulties in the estimation of the parameter variance-covariance matrix (Garre & Vermunt, 2006; Vermunt & Magidson, 2004), the sampling distribution of **ϑ** is approximated through nonparametric bootstrap (Efron & Tibshirani, 1994). A schematic of the MI procedure is presented in Fig. 1. The proposed procedure is here applied to the τ_*k*_ and τ indices, although it should offer comparable results with other indicators of classification reliability. The multiple imputation procedure for correcting τ_*k*_ and τ can be readily implemented using the *R* codes available at https://osf.io/cwfqx. Additionally, these code will be included in the cdmTools (Nájera et al., 2022) *R* package version 1.0.3 within the function named *CA.MI()*.

## Simulation study

A simulation study was conducted to compare the proposed multiple imputation procedure, accounting for the uncertainty of the model parameters, with the traditional reliability estimators computed using model parameters point estimates obtained with the EM algorithm.

### Method

The simulation study design is summarized in Table 2. As it can be observed, five between-group factors were manipulated (the attribute structure, the generating and fitted model, test length, sample size, and item quality). For *K* = 5 attributes with a uniform or higher-order structure, response data were generated and estimated using the reduced and saturated models, DINA and G-DINA, respectively, for *J* = 15 and 30 items. The CDM datasets were simulated and estimated using the *simGDINA* function from the *GDINA* package (W. Ma & de la Torre, 2020). Four sample sizes (*N* = 100, 200, 500, and 1000) and three item qualities (*IQ* = 1 – *P*(**0**) – *P*(**1**) = 0.4, 0.6, and 0.8) were considered, where *P*(**0**), i.e., the guessing parameter, and *P*(**1**), i.e., the slip parameter, were *P*(**0**) = *P*(**1**) = 0.1, 0.2, and 0.3, for *IQ* = 0.4, 0.6, and 0.8, respectively. The guessing and slip probabilities denote the success probabilities for examinees mastering none or all attributes required by the item, respectively. For each of the 2 × 2 × 2 × 4 × 3 = 96 between group conditions, 100 replications were conducted. Both τ_*k*_ and τ indices were considered, and two estimation methods (i.e., with posterior distributions computed using EM point estimates, or with the MI procedure) were used with each simulated dataset and treated as a within-group factor. Finally, the *Q*-matrices simulated in this study are presented in Table 3.

**Table 2:** Summary of the simulation design

Between-group factor	Levels
Attribute structure	Uniform, higher-order
Generated and fitted model	DINA, G-DINA
Test length (*J*)	15, 30
Sample size (*N*)	100, 200, 500, 1000
Item quality (*IQ*)^*^	0.4, 0.6, 0.8
Within-group factor	Levels
Method	τ (τ_*k*_) with MI, τ (τ_*k*_) with EM point estimates

^*^IQ = 1 – *P*(**0**) – *P*(**1**), where *P*(**0**) and *P*(**1**) denote the success probabilities for examinees mastering none or all attributes required by the item, respectively

**Table 3:** Q-matrix for the simulation study with 30 items

Item #	*α*_1_	*α*_2_	*α*_3_	*α*_4_	*α*_5_	Item #	*α*_1_	*α*_2_	*α*_3_	*α*_4_	*α*_5_
1	1	0	0	0	0	16	0	1	0	1	0
2	0	1	0	0	0	17	0	1	0	0	1
3	0	0	1	0	0	18^*^	0	0	1	1	0
4	0	0	0	1	0	19	0	0	1	0	1
5	0	0	0	0	1	20^*^	0	0	0	1	1
6^*^	1	0	0	0	0	21^*^	1	1	1	0	0
7^*^	0	1	0	0	0	22	1	1	0	1	0
8^*^	0	0	1	0	0	23^*^	1	1	0	0	1
9^*^	0	0	0	1	0	24	1	0	1	1	0
10^*^	0	0	0	0	1	25	1	0	1	0	1
11^*^	1	1	0	0	0	26^*^	1	0	0	1	1
12	1	0	1	0	0	27^*^	0	1	1	1	0
13	1	0	0	1	0	28	0	1	1	0	1
14^*^	1	0	0	0	1	29	0	1	0	1	1
15^*^	0	1	1	0	0	30^*^	0	0	1	1	1

* Asterisks* indicate the items in the *J* = 15 condition

Whereas the success probabilities for examinees mastering none or all attributes required by the items were defined by design (*IQ*), the remaining item parameters in the G-DINA model were drawn from a uniform distribution, constrained to monotonically increase with the number of attributes they are associated with, e.g., *P*(1,1) > *P*(1,0) > *P*(0,0). Additionally, under the higher-order attribute structure, *N* continuous latent factor scores, i.e., ${\mathit{\mathsf{\theta}}}_{\mathit{\mathsf{i}}}$, were drawn from *N*(0, 1), and the probability of mastering each attribute was computed as a two-parameter logistic model (Eq. 9) with ${\mathit{\mathsf{a}}}_{\mathit{\mathsf{k}}}=1.5$ and ${\mathit{\mathsf{b}}}_{\mathit{\mathsf{k}}}=0$ for every attribute. These values imply an expected prevalence of 0.5 for each attribute and an expected attribute intercorrelation of approximately 0.3. The true discrete attribute classifications for each simulee *i* and attribute *k* were then drawn from a binomial distribution with $P\left({\mathit{\mathsf{\alpha}}}_{\mathit{\mathsf{i}\mathsf{k}}}=1|{\mathit{\mathsf{\theta}}}_{\mathit{\mathsf{i}}},{\mathit{\mathsf{a}}}_{\mathit{\mathsf{k}}},{\mathit{\mathsf{b}}}_{\mathit{\mathsf{k}}}\right)$.

9 $$P\left({\alpha}_{ik}=1|{\theta}_i,{a}_k,{b}_k\right)=\frac{1}{1+\mathit{\exp}\left[-{a}_k\left({\theta}_i-{b}_k\right)\right]}$$

It should be noted that the assessment conditions associated with greater sampling errors (e.g., smaller sample sizes, lower item quality, more complex models) should imply a greater variability in the model parameter estimates from one bootstrap resample to another. Therefore, these conditions may require a larger number of resamples to provide stable MI reliability estimates. To address this, a preliminary simulation study was done to determine the acceptable number of resamples to use in the bootstrapping. This simulation was conducted by estimating τ with the MI procedure (τ^MI^) 50 times for one generated dataset under each assessment condition while manipulating the number of resamples within the bootstrap (*R* = 100, 200, 500, and 1000). As a criterion, the standard deviations (*SD*) of the τ^MI^ estimates for each dataset and *R* condition were analyzed. The overall results of this simulation can be found in Figure S1 (in the online supplementary material available at https://osf.io/cwfqx). In general, all the simulated *R* conditions provided sufficiently stable τ^MI^ estimates even in the most challenging assessment conditions. For instance, the largest *SD* (i.e., 0.01, for sample size of 100, bad item quality, with uniform attribute structure, and using the G-DINA model) indicates a narrow 95% confidence interval, between approximately 0.02 below and 0.02 above the average τ^MI^ estimate. In this article, nonetheless, a conservative *R* of 500 was used to reduce the effect of these estimation errors in the comparison between the different τ estimation methods. If needed, practitioners might use smaller number of resamples (e.g., *R* = 100) without a big loss of precision in the estimation of τ^MI^.

#### Data analysis

Under each condition, the simulees’ attribute profiles were estimated using EAP with the MMLE of the model parameters from the corresponding sample. The accuracy indices ${\uptau}_k^{\mathrm{EM}}$ and τ^EM^ of these attribute profiles were calculated as in Eqs. (6) and (7) with $P\left({\hat{\upalpha}}_{ik}|{\mathbf{x}}_n,\boldsymbol{\updelta}, \boldsymbol{\uppi} \right)$ computed using the point estimates of $\hat{\boldsymbol{\updelta}}$ and $\hat{\boldsymbol{\uppi}}$ obtained with the EM algorithm. Alternatively, ${\uptau}_k^{\mathrm{MI}}$ and τ^MI^ refer to the reliability indices calculated with the proposed multiple-imputation procedure (as outlined in Fig. 1), integrating $\hat{\boldsymbol{\updelta}}$ and $\hat{\boldsymbol{\uppi}}$ out through multiple-imputation. As a benchmark, the average true classification accuracy values (i.e., PCA and PCV) were calculated using the proportion of correct classification for the attribute profiles in each sample, given the known generated profiles. The average PCA and PCV consist of the expected true accuracy for the estimated attribute profiles under each condition. Additionally, to summarize the results, the generalized eta-square (${\eta}_G^2$) effect sizes of each manipulated factor over the absolute difference of τ (τ_*k*_) with respect to the average PCV (PCA) were computed through in mixed-effects ANOVAs. Finally, the root-mean-square error (RMSE; Eq. 10) was calculated between each τ (τ_*k*_) estimate and the average PCV (PCA) in every condition (and averaged across *K* for τ_*k*_).

10 $$\mathrm{RMSE}\left(\hat{\uptau}\right)=\sqrt{\frac{\sum_{r=1}^R{\left({\hat{\uptau}}_r- PCV\right)}^2}{R}}$$

### Results

Due to space limitations, only the results concerning the accuracy estimates at the attribute profile level (τ) and the uniform attribute structure conditions are presented in this document. In this regard, as will be detailed further, the effects of the manipulated factors over τ were largely generalizable to τ_*k*_. Similarly, the higher-order attribute structure condition, despite providing overall better results, did not substantially differ from the uniform attribute structure regarding the absolute error of τ and τ_*k*_. Therefore, the results for τ_*k*_ estimation and higher-order attribute structure are presented in the online supplementary material available at https://osf.io/cwfqx.

Table 4 presents the mean $\hat{\uptau}$ estimates along with the mean PCV for each condition. As could be expected, under each *IQ* and *J* condition, the PCV generally increased with sample size. This indicates that, regardless of the true test quality (i.e., given the true item parameters), classifications may be less accurate with smaller samples due to the inaccuracy of the model parameter estimates. As sample size increases, the PCV (and PCA) tends towards its highest value, which would be obtained if the true model parameters were known. Additionally, it can be observed that the ${\hat{\uptau}}^{\mathrm{MI}}$ estimates were consistently closer to the PCV in most conditions, whereas ${\hat{\uptau}}^{\mathrm{EM}}$ systematically overestimated it. In particular, the advantage of the proposed multiple-imputation method was evident under the most challenging estimation conditions (i.e., smaller sample size, saturated model, and low item quality). A slight overestimation was found for ${\hat{\uptau}}^{\mathrm{MI}}$ when the true reliabilities were very low (e.g., PCV = 0.13 and ${\hat{\uptau}}^{\mathrm{MI}}$ = 0.30 for *IQ* = 0.4, *N* = 100, *J* = 15, and G-DINA model). Nonetheless, from the author’s perspective, this overestimation was not sufficient to wrongly assume that the reliability was good for decision-making, as it generally occurred with ${\hat{\uptau}}^{\mathrm{EM}}$ (i.e., ${\hat{\uptau}}^{\mathrm{EM}}$ was equal to 0.87 in that specific condition).

**Table 4:** Average classification accuracy estimates at the profile level with uniform attribute structure

		G-DINA						DINA					
		*J* = 15			*J* = 30			*J* = 15			*J* = 30		
*IQ*	*N*	PCV	${\hat{\uptau}}^{\mathrm{EM}}$	${\hat{\uptau}}^{\mathrm{MI}}$	PCV	${\hat{\uptau}}^{\mathrm{EM}}$	${\hat{\uptau}}^{\mathrm{MI}}$	PCV	${\hat{\uptau}}^{\mathrm{EM}}$	${\hat{\uptau}}^{\mathrm{MI}}$	PCV	${\hat{\uptau}}^{\mathrm{EM}}$	${\hat{\uptau}}^{\mathrm{MI}}$
0.4 (Low)	100	0.13	0.87	0.30	0.20	0.92	0.28	0.15	0.69	0.29	0.26	0.72	0.33
	200	0.12	0.75	0.24	0.19	0.81	0.23	0.16	0.59	0.28	0.28	0.59	0.34
	500	0.12	0.59	0.19	0.21	0.62	0.23	0.17	0.42	0.27	0.33	0.46	0.37
	1000	0.13	0.47	0.19	0.25	0.49	0.26	0.19	0.33	0.27	0.35	0.41	0.39
0.6 (Medium)	100	0.28	0.86	0.39	0.49	0.92	0.48	0.37	0.71	0.48	0.62	0.81	0.62
	200	0.28	0.75	0.34	0.52	0.82	0.50	0.39	0.60	0.47	0.65	0.74	0.65
	500	0.31	0.59	0.34	0.60	0.70	0.58	0.42	0.52	0.47	0.68	0.70	0.68
	1000	0.34	0.50	0.37	0.62	0.67	0.63	0.44	0.48	0.46	0.68	0.70	0.69
0.8 (High)	100	0.58	0.90	0.59	0.85	0.97	0.76	0.69	0.83	0.70	0.90	0.94	0.84
	200	0.60	0.82	0.60	0.87	0.93	0.83	0.71	0.78	0.72	0.91	0.93	0.89
	500	0.64	0.73	0.65	0.89	0.91	0.89	0.72	0.75	0.73	0.91	0.92	0.91
	1000	0.67	0.71	0.68	0.90	0.90	0.90	0.72	0.74	0.73	0.91	0.92	0.91

*IQ* item quality, *N* sample size, *J* test length, *PCV* true classification accuracy, ${\hat{\uptau}}^{\mathrm{MI}}$ multiple imputation-based τ estimator, ${\hat{\uptau}}^{EM}$ EM point estimates-based τ estimator

Table 5 presents the effect sizes of the manipulated factors in a mixed-effects ANOVAs. The interactions with small or medium effects (${\eta}_G^2$ < 0.14) were omitted, and the complete table can be found in the online supplementary material (https://osf.io/cwfqx). As previously mentioned, no large effect sizes were observed for the attribute structure factor.

**Table 5:** Generalized Eta-squared for mixed-effects ANOVA of absolute reliability estimation error at the profile and attribute levels (*τ* and *τ*_*k*_, respectively)

	$\hat{\uptau}$	${\hat{\uptau}}_k$
Within-group effects		
Method	0.90^*^	0.84^*^
Method × Model	0.65^*^	0.47^*^
Method × *J*	0.22^*^	0.18^*^
Method × *N*	0.70^*^	0.58^*^
Method × *IQ*	0.78^*^	0.78^*^
Method × Model × *IQ*	0.34^*^	0.31^*^
Method × *N* × *IQ*	0.34^*^	0.36^*^
Between-group effects		
Model	0.65^*^	0.56^*^
Att. Struct.	0.06^*^	0.13^*^
*J*	0.54^*^	0.65^*^
*N*	0.83^*^	0.80^*^
*IQ*	0.86^*^	0.91^*^
Model × N	0.19^*^	0.06^*^
Model × IQ	0.27^*^	0.20^*^
*J × IQ*	0.08^*^	0.23^*^
*N × IQ*	0.40^*^	0.52^*^

Method = reliability estimation method (MI or EM-based); Model = generated/fitted model (DINA and G-DINA); Att. Struct = attribute structure; *IQ* = item quality; *N* = sample size; *J* = test length; ^*^ *p* < 0.05. Interactions with small or medium effects (${\eta}_G^2<0.14$) for both $\hat{\uptau}$ and ${\hat{\uptau}}_k$ are omitted

**Fig. 2:**
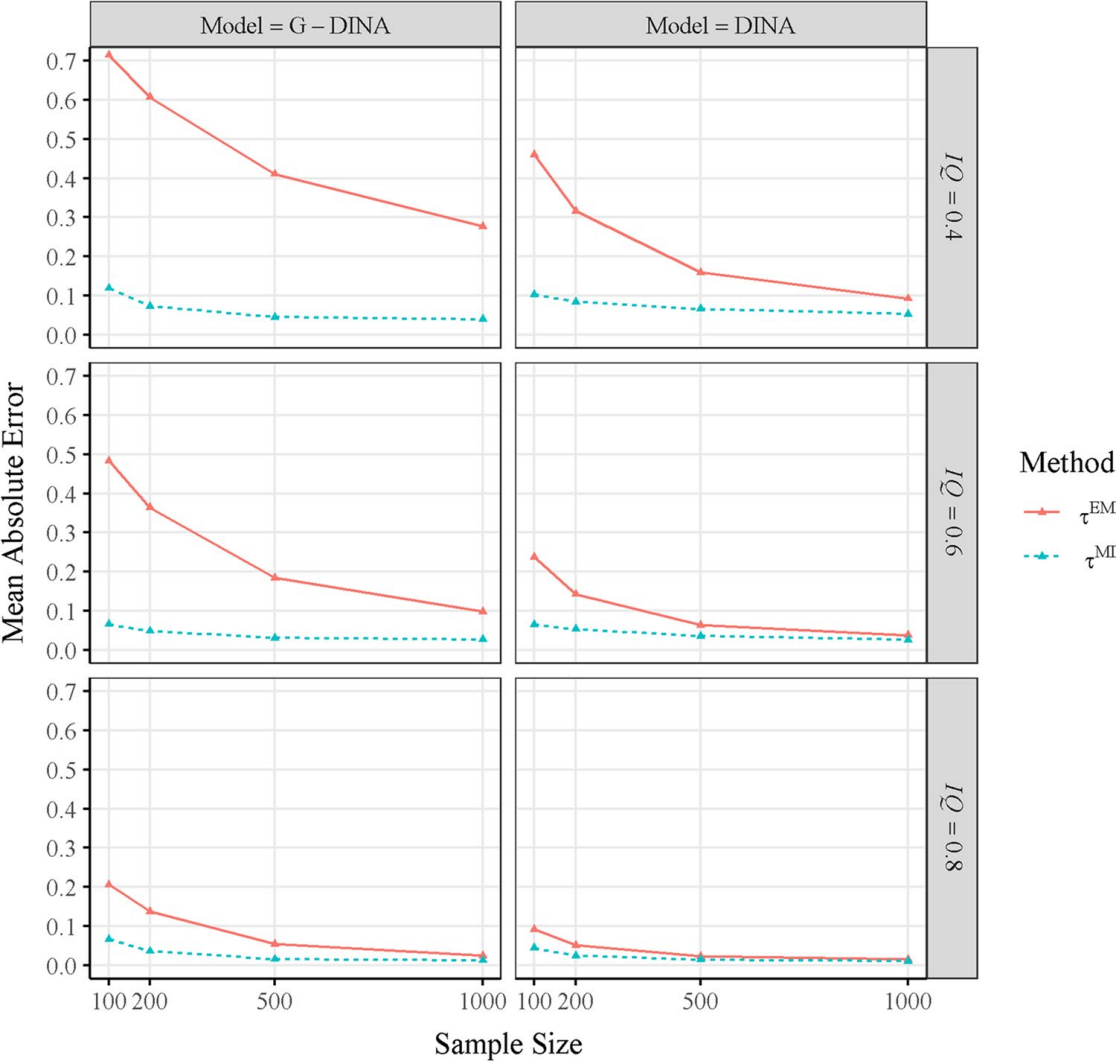
Means absolute errors of ${\hat{\boldsymbol{\uptau}}}^{\mathbf{EM}}$ and ${\hat{\boldsymbol{\uptau}}}^{\mathbf{MI}}$ for different sample sizes, item qualities, and generating and fitted models**.**  ${\hat{\boldsymbol{\uptau}}}^{\mathbf{MI}}$ = absolute error of the multiple imputation-based **τ** estimator; ${\hat{\boldsymbol{\uptau}}}^{\mathbf{EM}}$ = absolute error of the EM point estimates-based **τ** estimator

Figure 2 accounts for the major interaction effects found in Table 5 (i.e., with Method, Model, *IQ*, and *N*). It presents the means absolute errors of ${\hat{\uptau}}^{\mathrm{EM}}$ and ${\hat{\uptau}}^{\mathrm{MI}}$ for the different sample sizes, item qualities, and generating and fitted models. As can be observed, the differences between the two τ estimation methods were largely due to the variability of the absolute errors of ${\hat{\uptau}}^{\mathrm{EM}}$ in the simulated conditions, whereas the absolute errors of ${\hat{\uptau}}^{\mathrm{MI}}$ were only slightly affected by the manipulated factors. Specifically, the appropriateness of ${\hat{\uptau}}^{\mathrm{EM}}$ was largely affected by both sample size and item quality, and these effects were even greater when the generating and fitted model was the G-DINA. In this sense, the accuracy of the model parameter estimates is expectedly lower with smaller sample sizes, the G-DINA model (i.e., with higher number of parameters), and lower item qualities. Accordingly, ignoring the model parameter uncertainty with ${\hat{\uptau}}^{\mathrm{EM}}$ in these conditions provided overestimated reliabilities.

Table 6 presents the RMSE of ${\hat{\uptau}}^{\mathrm{MI}}$ and ${\hat{\uptau}}^{\mathrm{EM}}$ with respect to the PCV. Consistent with the results in Table 4, using ${\hat{\uptau}}^{\mathrm{EM}}$ provided inaccurate results, especially for the G-DINA model, with tests of lower item qualities, smaller samples, and lesser items.

**Table 6:** Root-mean-square error of MI and EM-based classification accuracy estimators at the profile level with uniform attribute structure

		G-DINA				DINA			
		*J* = 15		*J* = 30		*J* = 15		*J* = 30	
*IQ*	*N*	${\hat{\uptau}}^{\mathrm{EM}}$	${\hat{\uptau}}^{\mathrm{MI}}$	${\hat{\uptau}}^{\mathrm{EM}}$	${\hat{\uptau}}^{\mathrm{MI}}$	${\hat{\uptau}}^{\mathrm{EM}}$	${\hat{\uptau}}^{\mathrm{MI}}$	${\hat{\uptau}}^{\mathrm{EM}}$	${\hat{\uptau}}^{\mathrm{MI}}$
0.4 (Low)	100	0.73	0.17	0.72	0.10	0.54	0.15	0.46	0.09
	200	0.63	0.12	0.62	0.06	0.43	0.14	0.31	0.08
	500	0.47	0.08	0.40	0.03	0.25	0.11	0.13	0.05
	1000	0.34	0.07	0.25	0.03	0.15	0.09	0.07	0.04
0.6 (Medium)	100	0.58	0.11	0.43	0.05	0.34	0.12	0.19	0.04
	200	0.47	0.08	0.30	0.04	0.21	0.09	0.10	0.03
	500	0.29	0.05	0.10	0.03	0.10	0.06	0.03	0.02
	1000	0.16	0.04	0.05	0.02	0.05	0.03	0.02	0.01
0.8 (High)	100	0.32	0.06	0.11	0.10	0.14	0.04	0.05	0.06
	200	0.22	0.05	0.07	0.04	0.08	0.03	0.02	0.02
	500	0.09	0.03	0.02	0.01	0.04	0.02	0.01	0.01
	1000	0.05	0.03	0.01	0.01	0.02	0.02	0.01	0.01

*IQ* item quality, *N* sample size, *J* test length, ${\hat{\uptau}}^{\mathrm{MI}}$ multiple imputation-based τ estimator, ${\hat{\uptau}}^{\mathrm{EM}}$ EM point estimates-based τ estimator

As a general summary of the simulation results, the reliability estimates using the MI procedure to account for the uncertainty of the model parameters were almost always more accurate than their counterpart using EM-based model parameter point estimates. Specifically, the reliability estimation using point estimates was often overly positive, especially with low sample sizes and worse assessment conditions (i.e., lower-quality items, shorter tests). This can be alarming, since this overconfidence provided by ${\hat{\uptau}}^{\mathrm{EM}}$ can lead to making incautious decisions in especially delicate settings (with low true reliability). For instance, in the results for *N* = 100, *IQ* = 0.4, G-DINA model, and *J* = 30, presented in Table 4 an average ${\hat{\uptau}}^{\mathrm{EM}}$ of 0.92, indicating that 92% of respondents are expected to be correctly classified, when the actual correct classification was around 20%.

## Real data illustration

A study was conducted to compare ${\hat{\uptau}}^{\mathrm{EM}}$ and ${\hat{\uptau}}^{\mathrm{MI}}$ with a real dataset. The effects of sample size were studied by resampling subsets of response vectors from the complete data.

### Method

#### Data description

This study includes response data of 2922 examinees to the grammar section of the *Examination for the Certificate of Proficiency in English* (ECPE), as in Templin and Hoffman (2013). The ECPE was developed by the English Language Institute of the University of Michigan to assess a set of language skills for speakers of English as a non-primary language. The ECPE data have been already investigated from a diagnostic perspective in several studies (e.g., Akbay & de la Torre, 2020; Feng et al., 2014; Templin & Bradshaw, 2014). The grammar section of the test is composed of 28 multiple-choice items in which examinees are instructed to select the word, among four alternatives, that correctly fills the blank in a sentence. The grammar section of the ECPE measures three attributes, being the knowledge of (1) morphosyntactic rules, (2) cohesive rules, and (3) lexical rules (Buck & Tatsuoka, 1998). The Q-matrix for the 28 items, as defined in Templin and Hoffman (2013), are presented in Table 7. Also, accordingly with Templin and Hoffman (2013) and Akbay and de la Torre (2020) a saturated model (i.e., G-DINA) was fitted in this study. The ECPE response data and Q-matrix are available in the *GDINA* package (W. Ma & de la Torre, 2020) in *R* software environment.

**Table 7:** Q-matrix for the ECPE data (as in Templin & Hoffman, 2013)

Item #	Skill 1	Skill 2	Skill 3	Item #	Skill 1	Skill 2	Skill 3
1	1	1	0	15	0	0	1
2	0	1	0	16	1	0	1
3	1	0	1	17	0	1	1
4	0	0	1	18	0	0	1
5	0	0	1	19	0	0	1
6	0	0	1	20	1	0	1
7	1	0	1	21	1	0	1
8	0	1	0	22	0	0	1
9	0	0	1	23	0	1	0
10	1	0	0	24	0	1	0
11	1	0	1	25	1	0	0
12	1	0	1	26	0	0	1
13	1	0	0	27	1	0	0
14	1	0	0	28	0	0	1

Skill 1 = Morphosyntactic rules; Skill 2 = Cohesive rules; Skill 3 = Lexical rules

#### Procedures

In accordance with the previous simulation study, four sample size conditions (*N* = 100, 200, 500, and 1000) were manipulated. One hundred reduced samples for each size *N* were created by resampling *N* response vectors without replacement from the complete sample. The G-DINA model was fitted in each resampled dataset and the attribute profiles of the *N* respondents were estimated using EAP. The accuracy of these attribute profiles was computed using both ${\hat{\uptau}}^{\mathrm{MI}}$ and ${\hat{\uptau}}^{\mathrm{EM}}$ and the scatterplots between ${\hat{\uptau}}^{\mathrm{MI}}$ and ${\hat{\uptau}}^{\mathrm{EM}}$ in each condition are presented. As benchmark (dashed lines in the scatterplots), an approximation to the average true accuracy of the estimated attribute profiles (i.e., $\hat{\boldsymbol{\upalpha}}$) was done by averaging across the 100 replications the $\hat{\uptau}$ computed with the posterior probabilities obtained with the model parameter estimates from the complete dataset, i.e., $P\left({\hat{\boldsymbol{\upalpha}}}_i|{\mathbf{x}}_i,\hat{\boldsymbol{\updelta}},\hat{\boldsymbol{\uppi}}\right)$, where $\hat{\boldsymbol{\updelta}}$ and $\hat{\boldsymbol{\uppi}}$ were estimated with the 2922 examinees. In this sense, these model parameters obtained with the complete sample may be assumed to be largely precise, as ${\hat{\uptau}}^{\mathrm{MI}}$ and ${\hat{\uptau}}^{\mathrm{EM}}$ largely coincide in this condition (${\hat{\uptau}}^{\mathrm{MI}}=0.742$ and ${\hat{\uptau}}^{\mathrm{EM}}=0.743$). The same procedure was conducted for ${\hat{\uptau}}_k^{MI}$ and ${\hat{\uptau}}_k^{EM}$, and the results are presented in the online supplementary material.

### Results

**Fig. 3:**
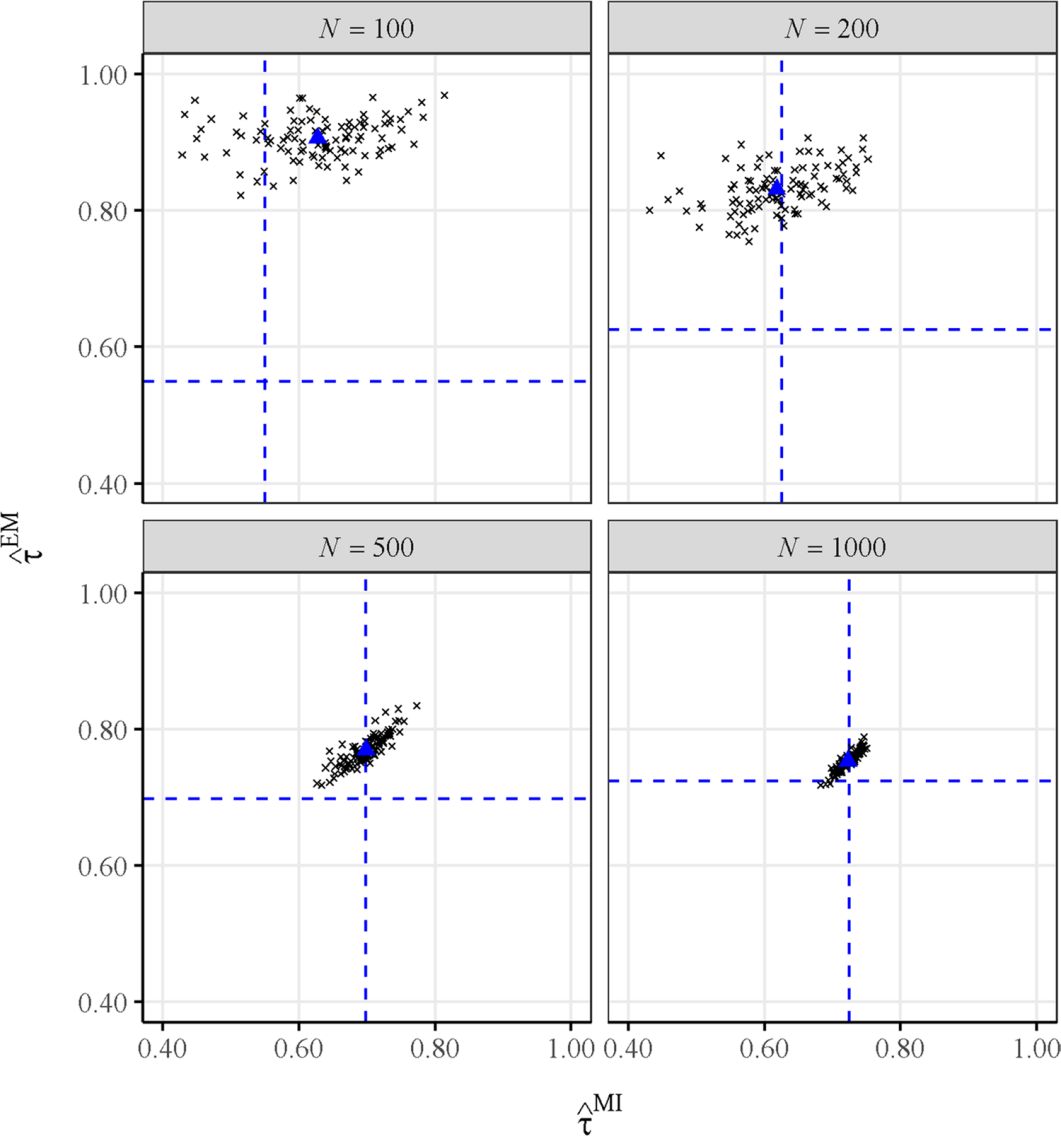
Dispersion between ${\hat{\tau}}^{EM}$ and ${\hat{\tau}}^{MI}$ of subsamples from the ECPE dataset by sample size (*N*). The *dashed lines* approximate the true reliability of the estimated attribute profiles in each sample size condition (using posteriors from the 2992-examinee sample). The *triangle* represents the average across the 100 replications in each condition

The G-DINA model provided generally good absolute fit with the complete data (M2 = 507.1459 with *df* = 325, RMSEA2 = 0.014, and SRMSR = 0.032). Figure 3 presents the dispersion between ${\hat{\uptau}}^{\mathrm{MI}}$ (*x*-axis) and ${\hat{\uptau}}^{\mathrm{EM}}$ (*y*-axis) estimates over 100 replications as a function of the size of the subsamples extracted from the ECPE dataset (panels). For instance, the top-left panel in Fig. 3 represents the dispersion between ${\hat{\uptau}}^{\mathrm{MI}}$ and ${\hat{\uptau}}^{\mathrm{EM}}$ computed using the model parameters estimated with the 100-examinee subsample. Due to the wide variance of the model parameter estimators in this condition, assuming the point estimates as true values in ${\hat{\uptau}}^{\mathrm{EM}}$ led to a large overestimation of the reliability. As the calibration sample size increases, the errors in the model parameter estimates tend to reduce, as does the effect of assuming point estimates as true in ${\hat{\uptau}}^{\mathrm{EM}}$. Therefore, in agreement with the simulation results, ${\hat{\uptau}}^{\mathrm{EM}}$ was especially overestimated for the smaller sample sizes, with *N* of 100 and 200. With higher sample sizes (i.e., 1000), both ${\hat{\uptau}}^{\mathrm{EM}}$ and ${\hat{\uptau}}^{\mathrm{MI}}$ tended towards the accuracy computed with the model parameters from the complete sample. Similar results have been found for the classification accuracy at the attribute level (i.e., ${\hat{\uptau}}_k$), which are presented in the online supplementary material (https://osf.io/cwfqx). Additionally, as in the simulation study, the average true accuracy approximations (dashed lines) were lower for the attribute profiles estimated from smaller samples. Specifically, the values of the dashed lines suggest that the attribute profiles estimated with the model parameters calibrated with small samples (e.g., 100) have low posterior probabilities (e.g., approximately 0.55) when using the model parameter estimates from the complete 2992-examinee sample. As sample size increases, the benchmark indicator converges towards the classification accuracy obtained with the complete dataset (approximately 0.74). As a summary, all the results with real data were consistent with the simulation study, where the classification accuracy computed with the MI procedure systematically provided the best estimates.

## General discussion

This article aimed to present a multiple imputation procedure to account for the uncertainty of the model parameters in CDM reliability estimation. As a general summary in both simulation and real data studies, the proposed method provided more accurate, less biased, reliability estimates than its traditional counterpart, using model parameter point estimates.

### Practical implications

The main practical implications of the two studies are presented. First, as an overall result, ${\hat{\uptau}}^{\mathrm{EM}}$ estimates were consistently positively biased, whereas ${\hat{\uptau}}^{\mathrm{MI}}$ was found to be closer to the true reliability. Second, especially with smaller samples, low item quality, more complex models (i.e., G-DINA), or lesser items, ${\hat{\uptau}}^{\mathrm{EM}}$ provided overly confident reliability estimates. Consequently, using ${\hat{\uptau}}^{\mathrm{EM}}$ in these situations may lead to making wrong decisions inadvertently. For instance, when diagnosing respondents, using ${\hat{\uptau}}^{\mathrm{EM}}$ could lead to the conclusion that classifications are sufficiently accurate when they are not. In turn, this can lead to important implications for the examinees. As an example, practitioners may be led to decide not to provide educational training for students in need, or to refuse treatment to patients that need to be treated. In this scenario, using ${\hat{\uptau}}^{\mathrm{MI}}$ is likely to provide more realistic reliability estimates, indicating that longer or better tests should be required in order to make such important decisions with safety. Under good testing conditions (i.e., large samples, high-quality items, simple models), however, both ${\hat{\uptau}}^{\mathrm{EM}}$ and ${\hat{\uptau}}^{\mathrm{MI}}$ were found to be largely precise, offering comparable results. In summary, practitioners in small educational or clinical settings should be aware that the reliability estimation using model parameter point estimates may be positively biased. Therefore, it is strongly recommended to use the multiple imputation procedure to account for the uncertainty around the model parameters. To facilitate the use of the new procedure, the *R* codes for estimating ${\hat{\uptau}}^{\mathrm{MI}}$ and ${\hat{\uptau}}_k^{\mathrm{MI}}$ were made available at https://osf.io/cwfqx and will be included in the cdmTools (Nájera et al., 2022) *R* package version 1.0.3 within the function named *CA.MI()*.

### Limitations and future directions

Some limitations of this study are acknowledged. First, as previously mentioned and evidenced in the simulation study, the model complexity played an important role in the overestimation of ${\hat{\uptau}}^{\mathrm{EM}}$. This may be explained by the fact that, opposed to reduced models that only account for the attribute main effects, more complex models (e.g., G-DINA), require the estimation of more item parameters (i.e., the attribute interaction effects) that are associated with more latent groups. Therefore, under the same sample size conditions, more complex models have fewer examinees per latent group and parameter. Moreover, with saturated models, the complexity of the Q-matrix (i.e., the number of attributes per item and, consequently, the number of latent groups per item) may affect even more the precision of the item parameter, and thus the reliability estimation (Sorrel et al., 2021). This factor (i.e., Q-matrix complexity) was not accounted for in this study, although it may be expected that the MI procedure would be even more preferable to using point estimates with more complex items. In this sense, considering the literature review conducted by Nájera et al. (2021a, b), the Q-matrices included in these studies were relatively simple (with mostly one-attribute items and up to ${K}_j^{\ast }$ = 2 with the ECPE data) to average (with one-third of one-attribute items and up to ${K}_j^{\ast }$ = 3 in the simulation study).

Second, as it has been thoroughly investigated in the recent literature (Gao et al., 2017; Rupp & Templin, 2008), model or Q-matrix misspecifications may largely affect the estimation classification accuracy. Consistently, although it was not addressed in this article, these misspecifications may also have an impact over the reliability estimation. In this sense, the proposed MI procedure may also be expected to perform better than its counterpart using EM point estimates, as the misspecifications may be partially captured by the standard errors of the item parameter estimates. Therefore, accounting for the sampling distribution of the item parameters may correct in part the wrong model or Q-matrix assumptions, which could be investigated in future studies.

Third, despite generally providing better reliability estimates, ${\hat{\uptau}}^{\mathrm{MI}}$ and ${\hat{\uptau}}_k^{\mathrm{MI}}$ were found to be slightly overestimated with small samples. This may be an indicator of the occurrence of boundary parameters within the bootstrapping procedure. As stated in the Introduction, these boundary parameters (i.e., 0 or 1 under identity link models) likely occur due to small sample sizes (Garre & Vermunt, 2006) or complex models leading to local maxima (Uebersax, 2000). Specifically, if models are complex and/or sample sizes are small, data are likely to be sparse (e.g., no observations in some latent groups, or only correct/incorrect responses for a given latent group), leading to boundary parameters. As stated by Garre and Vermunt (2006), using Bayesian estimation with non-informative priors may reduce such effect. Alternatively, other ad hoc approaches, such as fixing zero probabilities for unobserved latent groups in a sample may also reduce the boundary problem. This approach may be useful in case there are empirical zero counts for some latent groups, so that no inference should made around them in the sample. It would be interesting for future studies to empirically investigate this approach. On the contrary, if boundary parameters occur in the response probabilities (i.e., all the responses for a given latent group are the same), fixing these item parameters values would not be expected to reduce the reliability overestimation.

Fourth, although this article focused on two largely used CDM models, the effect of the MI over the reliability estimation should be tested with other CDM models (e.g., accounting for polytomous responses or polytomous attributes). Similarly, it was the authors’ decision to focus on the classification accuracy indicated provided by τ and τ_*k*_. Other classification accuracy and consistency may be investigated in the future. Nonetheless, most of the other indices also rely on the correctness of posterior probabilities, thus the MI procedure should also be expected to provide better results than using EM point estimates.
